# Mist1 Inhibits Epithelial-Mesenchymal Transition in Gastric Adenocarcinoma via Downregulating the Wnt/β-catenin Pathway

**DOI:** 10.7150/jca.59138

**Published:** 2021-06-01

**Authors:** Xin Xie, Zhangjian Zhou, Yongchun Song, Xin Zhang, Chengxue Dang, Hao Zhang

**Affiliations:** 1 Department of Surgical Oncology, The First Affiliated Hospital of Xi'an Jiaotong University, Xi'an, Shaanxi, 710061, China.; 2 Department of Oncology, The Second Affiliated Hospital of Xi'an Jiaotong University, Xi'an, Shaanxi, 710004, China.

**Keywords:** Mist1, gastric cancer, EMT, Wnt/β-catenin signalling pathway

## Abstract

As a secretory cell transcription factor, muscle intestine stomach expression 1 (Mist1) is associated with serous secretory cell development and gastric chief cell maturation. Here, we focus on the function of Mist1 in gastric adenocarcinoma carcinogenesis. Based on clinical data and a mouse model of gastric cancer, we found that Mist1 expression was reduced in gastric cancer. Then, we overexpressed Mist1 using a lentivirus system and found that overexpression of Mist1 could inhibit gastric cancer cell proliferation, migration and invasion *in vitro.* Additionally, *in vivo*, we assessed the function of Mist1 in a gastric cancer xenograft model and distant pulmonary metastasis model. Overexpression of Mist1 decreased tumour growth and distant metastasis *in vivo*, suggesting that Mist1 acts as a tumour suppressor in gastric carcinogenesis. Furthermore, Mist1 overexpression inhibited epithelial-mesenchymal transition (EMT) in gastric cancer by suppressing β-catenin transcription activity and then the Wingless and INT-1 (Wnt)/β-catenin signalling pathway, which could be reversed by a Wnt/β-catenin-specific agonist. In conclusion, this study indicated that overexpression of Mist1 could reverse EMT in gastric carcinogenesis by inhibiting the Wnt/β-catenin signalling pathway and that Mist1 might be a novel marker for early gastric cancer screening.

## Introduction

Gastric adenocarcinoma is a major kind of malignant tumour in the gastrointestinal system, and more than 1,000,000 new cases and approximately 783,000 deaths from gastric adenocarcinoma occurred worldwide in 2018 1. The incidence of gastric adenocarcinoma is predominantly elevated in Eastern Asian countries, including Japan, Korea, Mongolia and China. In China, gastric adenocarcinoma has become a heavy public health burden, with an incidence of 18.57/100,000 and mortality of 12.92/100,000 in 2015 2, 3. Surgery, chemotherapy and radiotherapy are major therapies for gastric adenocarcinoma; however, the prognosis of gastric adenocarcinoma and patient quality of life are not satisfactory 4, 5. Therefore, better therapeutic and screening strategies are urgently needed to improve the outcome of gastric adenocarcinoma.

Epithelial-mesenchymal transition (EMT) is an important and necessary event in cellular development and the metastasis of epithelial carcinomas, including gastric cancer, colorectal cancer, and breast cancer 6-8. Based on transcriptional repression and cadherin delocalization, the core elements of EMT are loss of the apical-basal polarity of epithelial cells and cell-cell adherence, which contributes to tumour cell detachment from the basement membrane and distant metastasis 9. These characteristic changes during EMT enable epithelial cells to gain more variable mesenchymal-like cell shapes and alter the expression of cell-cell junction proteins, such as the downregulation of E-cadherin and the upregulation of N-cadherin 9-11. In contrast, mesenchymal-epithelial transition (MET) is the reverse of EMT; following MET, cells show epithelial-like phenotypes and the increased expression of tight junction proteins, which is also critical to cancer carcinogenesis 12, 13.

Muscle intestine stomach expression 1 (Mist1), also named bHLHa15, is a secretory cell transcription factor that belongs to the basic helix-loop-helix superfamily 14. Mist1 is mainly expressed in serous secreted cells and essential for the development and maturation of secretory cells 15, 16. Secretory cells lacking Mist1 exhibit structural and functional defects, including alterations in cytoskeletal organization and cell polarity and improper cell-cell junction 15, 17. In the pancreas, loss of the acinar-restricted transcription factor Mist1 accelerates Kras-induced pancreatic intraepithelial neoplasia (PaIN) 18. Additionally, the expression of Mist1 results in a significant decrease in the proliferative potential of pancreatic acinar cells associated with the induced expression of p21 (CIP1/WAF1) 19. In the stomach, Mist1 expression is restricted to the chief cell compartment in the normal oxyntic mucosa, which is rare in established metaplastic lesions, and absent in intraepithelial neoplasia/dysplasia and various types of carcinomas, with the exception of the rare chief cell carcinoma 20. Therefore, we hypothesize that Mist1 is a tumour suppressor involved in gastric cancer initiation and development. In this study, we investigated the function of Mist1 in gastric cancer EMT and its potential roles in the Wingless and INT-1 (Wnt)/β-Catenin pathway.

## Materials and methods

### Cell lines and culture

A human gastric epithelial cell line (GES-1), human gastric cancer cell lines (MKN-45, MKN-28, HGC-27, AGS, NCI-N87, KATO-III) and human colorectal cancer cell line (SW620) were used in this study. MKN-28 and NCI-N87 cells were cultured as previously described 21. GES-1, MKN-45, HGC-27, AGS, NCI-N87, KATO-III and SW620 cells were obtained from the Type Culture Collection of the Chinese Academy of Science (Shanghai, China). STR profiles of cell lines were authenticated. GES-1, MKN-45, HGC-27, KATO-III and SW620 cells were cultured in RPMI-1640 medium (HyClone, Logan, UT, USA) supplemented with 10% foetal bovine serum (FBS, Gibco, Carlsbad, CA, USA), and AGS cells were cultured in F12 medium (HyClone) supplemented with 10% FBS (Gibco). Gastric cancer cell lines were cultured at 37 °C in a humidified atmosphere containing 5% CO_2_.

### Overexpression of Mist1 in gastric cancer cell lines

The Mist1 overexpression gastric cancer cell lines were established by lentiviral vectors. Transfer plasmids pEZ-Lv105-Mist1 (no GFP protein contained) and negative control with empty vector pEZ-Lv105 were purchased from GeneCopoeia, Inc. (Rockville, MD, USA). To produce each lentivirus, 3 μg transfer plasmids were co-transfected with 1.5 μg and 3 μg packing plasmids pMD2.G (Addgene #12259, Cambridge, MA, USA) and psPAX2 (Addgene #12260), respectively, into 70% confluence HEK293T cell cultured in 6-cm cell culture dish by using 50 μL PLUS Reagent^TM^ (Thermo Fisher Scientific, Waltham, MA, USA) and 30μL Lipofectamine 2000^ TM^ (Thermo Fisher Scientific). Gastric cancer cell lines HGC-27 and AGS were cultured in 6-cm dishes and infected directly with 200 μL lentivirus containing supernatant from HEK293T cell. After 24 h of infection, fresh culture medium containing 2 μg/mL puromycin was added to select successful infected cells. Alteration of Mist1 expression was assessed by qPCR and Western blotting.

### Clinical tissue specimens

Gastric cancer tissue and matched adjacent noncancerous tissue specimens from same patients were obtained from 30 patients with gastric cancer who underwent surgery at the First Affiliated Hospital of Xi'an Jiaotong University. No patients received any chemotherapy or radiotherapy before surgery. Tissue samples were separated into two groups. One group was frozen and immediately stored in liquid nitrogen; the other group was fixed in formalin and then embedded in paraffin for haematoxylin and eosin (HE) staining and immunohistochemistry (IHC). This study was approved by the Ethical Committee of the First Affiliated Hospital of Xi'an Jiaotong University. Written informed consent was obtained from all patients who participated in this study.

### RNA extraction and quantitative real-time PCR (qPCR)

Total cellular RNA and tissue specimen RNA were extracted using TRIzol reagent following the manufacturer's protocol (Invitrogen, Carlsbad, CA, USA). Reversed transcription was completed using a PrimeScript^TM^ RT reagent kit (Takara, Shiga, Japan), and qPCR was performed on a Bio-Rad^®^ CFX96 Touch^TM^ Real-Time PCR Detection System using SYBR Premix Ex Taq^TM^ Ⅱ(Takara) with the following conditions: step 1: 95 °C for 30s; step 2: 40 cycles of 95°C for 5s and 60 °C for 30s. The primers used for qPCR amplification were listed in Supplementary Table 1. Relative mRNA levels are expressed as the fold change relative to expression of the GAPDH gene and were normalized to the control group.

### Western blotting

For total protein extraction, cells were lysed on ice with RIPA buffer containing 1% PMSF and 2% protease inhibitor cocktails (Hat Biotech, China). For nuclear or cytoplasmic protein extraction, cells were lysed by Nuclear and Cytoplasmic Extraction Kit (CW Bio, China) following manufacturer's instruction. The protein concentration was quantified by bicinchoninic acid (BCA) assay (CW Bio). Samples containing equal amounts of protein were separated on 10% sodium dodecyl sulfate-polyacrylamide gel electrophoresis (SDS-PAGE) gels and then transferred to apolyvinylidene difluoride (PVDF) membrane. The PVDF membrane was blocked with 5% fat-free milk at room temperature for 2 h and incubated overnight at 4 °C with primary monoclonal antibodies targeting Mist1, E-cadherin, N-cadherin, β-catenin, Snail, TCF-4, c-Myc, Cyclin D1, GAPDH (CST, Danvers, MA, USA), Vimentin, MMP9 (Santa Cruz, Dallas, TX, USA) and Lamin B1 (Proteintech, China), followed by incubation with secondary antibody (Proteintech) at room temperature for 1.5 h. Immunoreactivity was detected with an electrochemiluminescence system (Millipore, Darmstadt, Germany).

### Immunofluorescence (IF)

Gastric cancer cells were plated in 24-well plates containing cover slips. When the cells reached 60-70% confluence, the media were removed, and the cells were fixed with 4% paraformaldehyde for 15 min. Permeabilized cells were treated with 0.1% Triton X-100 (Solarbio, China) for 10 min, followed by blocking with 1% bovine serum albumin (BSA, Hat Biotech, China) for 1h. Then, the cells were incubated with primary antibodies against Mist1, E-cadherin, N-cadherin, β-catenin (CST) and Vimentin (Santa Cruz) in blocking buffer at 4 °C overnight. Next, the cells were incubated with FITC- or TRITC-conjugated secondary antibody and DAPI (Proteintech) at room temperature for 2 h. Cover slips were sealed with the slides with fluorescence decay-resistant medium (Hat Biotech).

### Cell proliferation, migration, invasion and colony formation assays

To assess gastric cancer cell proliferation, cells were plated into 96-well plates and incubated for 1, 3, 5 and 7 days. Then, the CCK-8 assay was performed according to the manufacturer's instructions (Hat Biotech).

Wound-healing and Transwell migration assays were used to investigate gastric cancer cell migration. For the wound-healing assay, cells were plated into 6-well plates. When the cells reached 90% confluence, they were scratched with 200 μL pipette tips. The migration area was measured at 0, 24, and 48 h after scratching by ImageJ software 22. For the Transwell migration assay, approximately 1×10^4^ cells were mixed with 200 μL of medium containing 2% FBS and then plated in the upper chamber of a 24-well Transwell plate (Thermo Fisher Scientific), and 700 μL of medium containing 20% FBS was added into the lower chamber of the Transwell plate.

For the invasion assay, the upper chamber of the Transwell plate was embedded in Matrigel (Corning, NY, USA), and approximately 5×10^4^ gastric cancer cells in 200 μL of medium containing 2% FBS was added to the chamber. The lower chamber contained 700 μL of medium containing 20% FBS. For the Transwell migration and invasion assays, after 24 h of incubation, cells in the upper chamber were fixed with 4% paraformaldehyde, stained with crystal violet and counted.

To investigate the ability of gastric cancer cells to form colonies, approximately 500 cells were plated into 6-well plates in medium containing 10% FBS and incubated for 14 days. Then, the cells were fixed in 4% paraformaldehyde and stained with crystal violet.

### Luciferase reporter assay

To explore whether Mist1 could influence the transcription activity of β-catenin, 5000 cells were plated into 96-well plates. After starvation with FBS-free medium overnight, 0.2 μg TOPflash or FOPflash reporter genes (Addgene #12456, #12457) were co-transfected with 0.1μg Renilla TK-luciferase vector (Promega, China) into the cells by Lipofectamine 2000^TM^. After 48h of transfection, luciferase activity was measured by Dual-Luciferase Reporter Assay Kit (Promega) using a Glomax 96 Microplate Luminometer. The firefly luciferase activity was normalized by Renilla luciferase and the β-catenin driven transcription activity was measured by TOP/FOP luciferase ratio.

### *In vitro* treatment with the Wnt/β-Catenin activator CHIR-99021

Gastric cancer cells were plated in 6-cm cell dishes and then incubated with 5 μM CHIR-99021 (Selleckchem, Houston, TX, USA) for 72 h, with the medium changed every 24 h. Protein and RNA samples were extracted for further experiments.

### Development of an *in vivo* gastric carcinogenesis model

**Figure 1:**
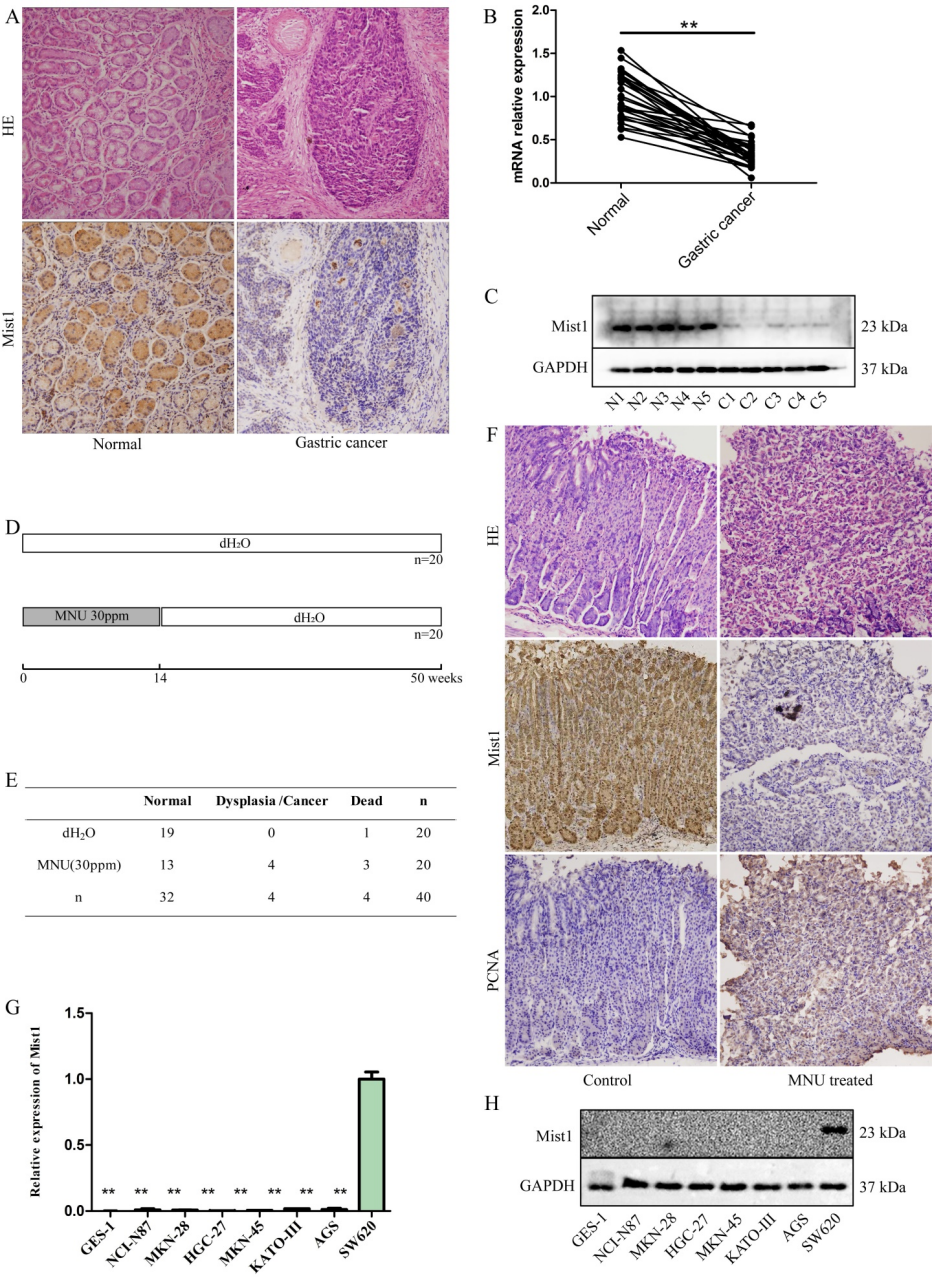
Mist1 expression is downregulated in gastric cancer tissue and cell lines. **A)** HE and IHC, **B)** qPCR and **C)** Western blotting assays for detecting Mist1 expression from clinical specimens, respectively. **D)** Schematic and **E)** survival status of MNU-induced gastric neoplasm model. **F)** HE and IHC assays for detecting Mist1 and PCNA expression in MNU treated mice model. **G)** qPCR and **H)** Western blotting assays for detecting Mist1 expression from gastric cancer cell lines. Human colorectal cancer cell SW620 is used as a positive control of Mist1. **P<0.01.

All protocols involving animal models or animal specimens were approved by the Ethical Committee of Xi'an Jiaotong University Medical College and the Experimental Animal Center of Xi'an Jiaotong University. The experimental schematic is shown in Fig. 1D. Forty C57BL/6 male mice were divided into two groups. N-Methyl-N-nitrosourea (MNU, Sigma, St. Louis, MO, USA) was dissolved in dH_2_O at a concentration of 30 p.p.m., and this solution was freshly prepared as drinking water twice per week. The drinking water was changed to normal dH_2_O on alternating weeks for 14 weeks, giving 7 total weeks of MNU exposure. Normal dH_2_O was used as the drinking water for the control group. All mice were euthanized by phenobarbitone (0.3 mg/10g body weight) at 50 weeks from the beginning of MNU administration. Gastric tissues were collected as previously described.

### Development of *in vivo* xenograft and pulmonary metastatic models

The HGC-27-Vector (NC) and HGC-27 Lv-Mist1 gastric cell lines were subcutaneously injected into the left flanks of BALB/c nude mice (4 weeks old, male) to develop a xenograft model or injected into the caudal vein to develop a pulmonary metastatic model. Each group consisted of five mice, and the xenograft tumour size was measured every 3 days. All mice were euthanized by phenobarbitone (0.3 mg/10g body weight) 28 days after injection.

### IHC

Formalin-fixed, paraffin-embedded tissue sample sections (thickness of 5 μm) were deparaffinized and rehydrated, followed by antigen retrieval with EDTA retrieval buffer (Hat Biotech) and blocking with 5% BSA (Hat Biotech). Sections were incubated with primary antibodies against Mist1 (1:100) and PCNA (1:100, CST) at 4 °C overnight. Then, the sections were incubated with secondary antibodies, stained with a 3,3'-diaminobenzidine (DAB) kit (ZSGB Bio, China) and counterstained with haematoxylin to visualize nuclei.

### Statistical analysis

All experiments were repeated at least 3 times and representative data was shown. Consecutive data are presented as the mean±standard deviation (SD). Differences between two groups were compared by Student's *t*-test or one-way analysis of variance (ANOVA). All statistical tests were 2-sided, and differences with *P<*0.05 were considered statistically significant. Statistical analyses were performed using R software version 3.3.2 (http://www.r-project.org).

## Results

### Mist1 is downregulated in gastric cancer and MNU-induced mouse gastric neoplasms

Given the important role of Mist1 in secretory cell development and maturation, we investigated the expression of Mist1 in gastric cancer tissue and matched adjacent normal tissue samples (n=30). IHC indicated that Mist1 expression was lower in gastric cancer tissue samples than in normal gastric tissue samples (Fig. 1A). Further validation was performed by extracting RNA and protein from both normal and gastric cancer tissue samples and detecting Mist1 expression by qPCR and Western blotting, respectively, which revealed that Mist1 is downregulated in the development of gastric cancer (Fig. 1B,C).

To validate expression changes of Mist1 in gastric carcinogenesis, we established an MNU-induced gastric neoplasm model in C57BL/6 mice (n=40, Fig. 1D). After 14 weeks of MNU administration and 50 weeks of observation, the 36 living mice were euthanized to assess gastric morphology and carry out IHC (dead mice: control group: 1, MNU-treated mice: 3, Fig. 1E). Among the remaining 17 mice in the MNU-treated group, 4 mice exhibited dysplasia or neoplasia (4/17, 23.53%) by HE staining. Consistent with clinical gastric cancer samples, Mist1 was downregulated in dysplasia and neoplasia specimens, while PCNA, a nuclear marker of proliferation, was significantly upregulated in comparison with its expression in the control group (Fig. 1F).

### Mist1 is downregulated in gastric cancer cell lines and effects of the overexpression of Mist1 *in vitro*

**Figure 2:**
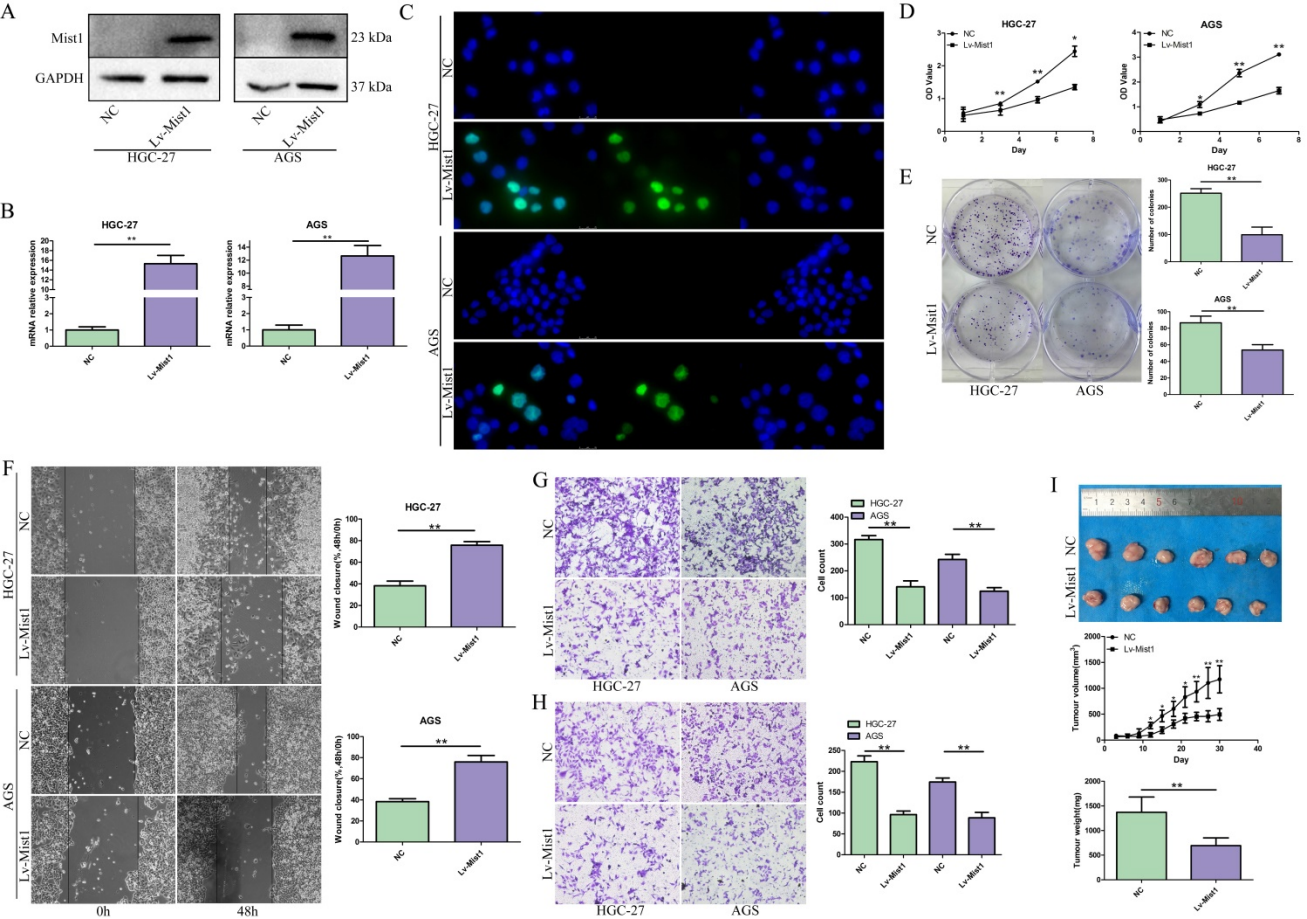
The impact of Mist1 overexpression in gastric cancer cells* in vitro* and* in vivo*. **A-C)** The construction of Mist1 overexpression in gastric cancer cell lines. The expression of Mist1 is detected by **A)** Western blotting, **B)** qPCR and **C)** IF assays (green stands for Mist1 inmmunostaining and blue for DAPI) respectively. Overexpression of Mist1 inhibits **D)** cell proliferation, **E)** colony formation, **F, G)** migration and **H)** invasion ability *in vitro*, and also **I)** tumour growth *in vivo*. *P<0.05, **P<0.01.

To confirm the expression of Mist1 in gastric cancer cells, we assessed seven gastric cell lines by qPCR and Western blotting, which indicated the downregulation of Mist1 expression in gastric cancer cells compared with SW620 cells, consistent with the changes in Mist1 expression in gastric cancer tissues (Fig. 1G, H). Thus, we hypothesized that Mist1 expression is downregulated during gastric carcinogenesis. Next, we overexpressed Mist1 in the HGC-27 and AGS gastric cell lines by transfection with the lentivirus system. As confirmed by qPCR, Western blotting and IF, Mist1 expression was significantly upregulated in HGC-27 Lv-Mist1 and AGS Lv-Mist1 transfected cells, in contrast to the negative control group, which was transfected with lentivirus containing empty vector (NC, Fig. 2A-C).

### Overexpression of Mist1 inhibits gastric cancer cell proliferation, colony formation, migration and invasion

To investigate the functions of Mist1 in gastric cancer cells, we overexpressed Mist1 in the HGC-27 and AGS gastric cancer cell lines. Proliferation was assessed by CCK-8 assay, which revealed that overexpressed Mist1 could significantly inhibit the proliferation of HGC-27 and AGS cells (Fig. 2D). Additionally, the colony formation ability of Mist1-overexpressing gastric cells was decreased (Fig. 2E). Wound-healing and Transwell migration assays indicated that the migratory ability of gastric cancer cells was reduced by the overexpression of Mist1 (Fig. 2F, G). Meanwhile, the results of the Transwell invasion assay indicated that Mist1 overexpression suppressed gastric cancer cell invasion* in vitro* (Fig. 2H).

To confirm the function of Mist1 in gastric cancer *in vivo*, we established a xenograft model by the subcutaneous injection of HGC-27 Lv-Mist1 cells or matched HGC-27 cells with empty vector as the NC group. After 4 weeks of observation, the volume and weight of tumours in the Mist1-overexpressing group were significantly decreased, indicating that Mist1 could inhibit gastric cancer growth *in vivo* (Fig. 2I).

### Overexpression of Mist1 inhibits EMT in gastric cancer cells via the Wnt/β-Catenin pathway

**Figure 3:**
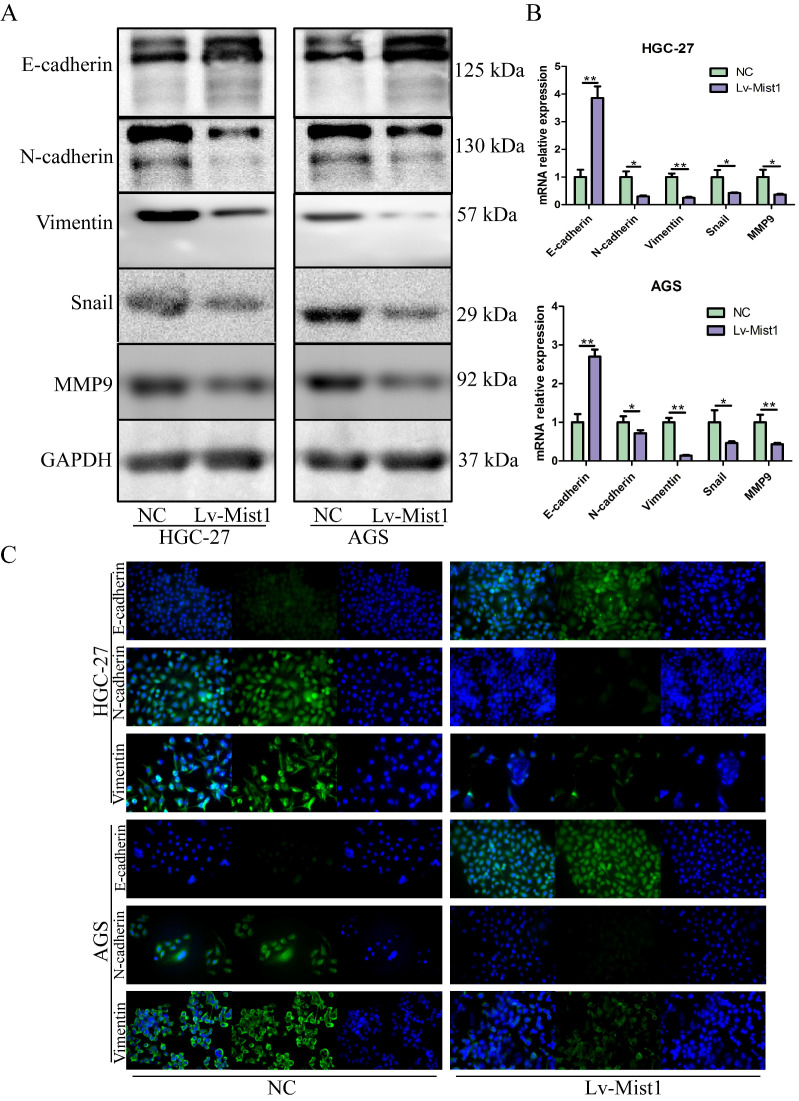
Mist1 overexpression inhibits EMT in gastric cancer cells. EMT is inhibited in Mist1 overexpression cells detected by **A)** Western blotting, **B)** qPCR and **C)** IF (green stands for E-cadherin, N-cadherin or Vimentin and blue stands for DAPI).

As an integral process in development, wound healing and stem cell behaviour, EMT also plays critical roles in gastric carcinogenesis and distant metastasis. Based on inhibitions of migration and invasion abilities in gastric cells by Mist1 overexpression, we further explored the relationship between Mist1 and EMT. Notably, the epithelial cell marker E-cadherin was upregulated in Mist1-overexpressing gastric cancer cells; however, the mesenchymal marker N-cadherin, Vimentin and E-cadherin repressor Snail were downregulated (Fig. 3A-C). Also, metastatic related gene, MMP9, was downregulated. Meanwhile, Mist1-overexpressing gastric cancer cells exhibited a slightly rounder shape compared with NC cells, which showed a spindle-like morphology (Supplementary Figure 1).

**Figure 4:**
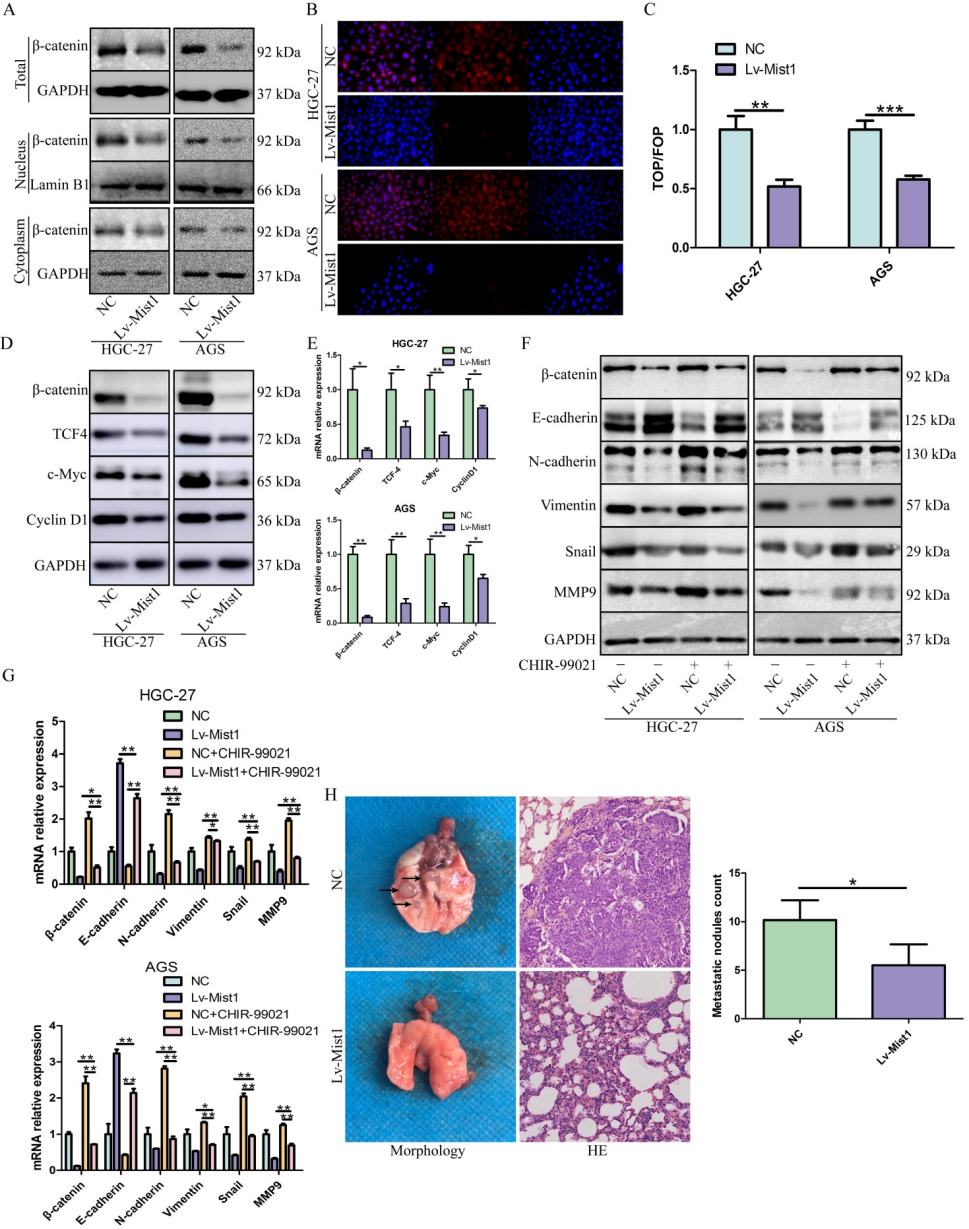
** Mist1 overexpression inhibits β-catenin transcription activity and the canonical Wnt/β-catenin pathway in gastric cancer cells. A)** Western blotting and **B)** IF showed β-catenin expression was decreased in both nucleus and cytoplasm (red stands for β-catenin and blue stands for DAPI). **C)** Luciferase assay indicated that β-catenin transcription activity was decreased in Mist1 overexpression cells. **D, E)** Mist1 overexpression downregulated the canonical Wnt/β-catenin pathway and** F, G)** the EMT inhibition was reversed by CHIR-99021. Mist1 overexpression decreases **H)** pulmonary metastasis *in vivo*. *P<0.05, **P<0.01, ***P<0.001.

Several studies have demonstrated the important roles of the Wnt signalling pathway in the processes of carcinogenesis, including cancer cell proliferation, metastasis and EMT. β-catenin is the core molecule of the canonical Wnt pathway, which regulates the activities of many downstream signals. In this study, we explored the expression of the canonical Wnt signalling pathway in Mist1-overexpressing gastric cancer cells and found that β-catenin was downregulated in Mist1-overexpressing cells. Furthermore, we found β-catenin expression was also downregulated in both nucleus and cytoplasm of Mist1-overexpression cells, indicating the transcription ability of β-catenin might be inhibited by Mist1 overexpression (Fig. 4A, B). To validate our hypothesis, TOP/FOP flash luciferase reporter assay was performed and showed decreased transcription activity of β-catenin in Mist1-overexpressing cells (Fig. 4C). In addition, we checked signalling molecules downstream of β-catenin and found that TCF-4, c-Myc and Cyclin D1 were reduced in Mist1-overexpressing cells, which indicates that the Wnt/β-catenin signalling pathway was inhibited in Mist1-overexpressing gastric cancer cells (Fig. 4D, E).

To further explore the potential relationship between Mist1 and the Wnt/β-catenin signalling pathway, we treated gastric cancer cells with the Wnt/β-catenin-specific agonist CHIR-99021. After 48h of administration, changes in β-catenin expression were reversed. Additionally, the expression of N-cadherin was upregulated, while E-cadherin expression was downregulated in Mist1-overexpressing cell lines, which revealed that Mist1 inhibited EMT through the Wnt/β-catenin signalling pathway (Fig. 4F, G).

### Overexpression of Mist1 decreases pulmonary metastasis *in vivo*

Distant metastasis causes poor prognosis in gastric adenocarcinoma. In this study, we explored whether the overexpression of Mist1 could inhibit pulmonary metastasis induced by gastric cancer cells *in vivo*. Compared with the NC group, the Mist1-overexpressing group exhibited significantly decreased pulmonary metastasis, suggesting that Mist1 suppresses distant metastasis in gastric cancer (Fig. 4H).

## Discussion

Gastric adenocarcinoma, a major type of gastric cancer, exhibits a high incidence and high mortality, especially in Eastern Asia, which brings a heavy economic and social burden to both patients and the public health system. Recent studies have emphasized that early diagnostic screening could be a better preventing strategy for gastric adenocarcinoma control 23-25. Unlike traditional screening by gastric endoscopy, a noninvasive screening strategy based on diagnostic biomarkers from the peripheral blood, urine or faeces might be a proper method for the early screening of gastric cancer. In this study, we investigated the expression status of Mist1 in gastric adenocarcinoma patients and its potential suppressive mechanism in gastric carcinogenesis via inhibition of the Wnt/β-catenin pathway, suggesting that the expression status of Mist1 is a potential screening or therapeutic target for gastric adenocarcinoma management.

As a secretory cell transcription factor, Mist1 is critical to serous secretory cell development and maturation. Secretory cells lacking Mist1 exhibit severe defects in cellular function and exocrine gland structure 26. During development of the pancreas, Mist1-knockout acinar cells were predisposed to a ductal phenotype, and epidermal growth factor receptor (EGFR) and Notch signalling pathways were activated 18. The expression of Mist1 in human gastric development and maturation has been described in recent years, and Mist1 has been suggested to regulate the maturation of exocrine granules via the RAB26/RAB3D complex and act a chief cell marker of gastric mucosa regeneration 17, 20, 27. Recently, an increasing number of studies have addressed the role of Mist1 in the carcinogenesis and development of pancreatic cancer, melanoma and salivary gland acinic cell carcinoma 28-30. Here, in our study, we found that Mist1 was downregulated in gastric carcinogenesis. Then, we overexpressed Mist1 in gastric cancer cells and found that overexpression of Mist1 could inhibit gastric cancer cell proliferation, migration and invasion both* in vitro* and *in vivo*, suggesting that Mist1 is a tumour suppressor gene in gastric adenocarcinoma initiation and development. Several studies have indicated that *Helicobacter pylori* (*H. pylori*) infection is found in half of the world's population and many genes are aberrantly hypermethylated in *H. pylori*-infected gastric mucosae 31-33. Thus, whether silent expression of Mist1 in gastric adenocarcinoma is related to aberrant methylation needs further exploration and investigation.

The Wnt signalling pathway has been found to be involved in many aspects of embryonic development and homeostatic self-renewal in a number of adult tissues since its discovery by Nusse and Varmus in 1982 34, 35. Several studies in recent decades have revealed that mutations in the Wnt signalling pathway are associated with hereditary diseases and a variety of cancers in different tissues, including the gastrointestinal tract 36-38. Wnt signalling regulates various cellular functions, such as cell proliferation, differentiation, and development and processes in disease progression, such as EMT 39, 40. Wnt target genes are thought to be activated by the activation of three different pathways: the canonical Wnt/β-catenin pathway, the noncanonial planar cell polarity (PCP) pathway and the Wnt/Ca^2+^ pathway 41. β-Catenin, the core of the canonical Wnt/β-catenin pathway, plays two roles in simple epithelia; it acts as a binding partner for adherens junction proteins, such as E-cadherin, or as a messenger in the signalling pool. Unlike the β-catenin signalling pool, which is rapidly destroyed by phosphorylation due to GSK-3α/β, β-catenin binds adherens junctions and is highly stable 35. Interestingly, the interaction between adhesion and signalling remains controversial. When Wnt signalling is activated, the Axin/GSK-3/CK1 complex disaggregates and stabilizes the β-catenin signalling pool. Then, stable β-catenin anchors the TCF/LEF complex to transcriptionally activate downstream genes 41. In colorectal cancer, the β-catenin/TCF4 complex regulates the expression of EphB/EphrinB, which is upregulated in early adenomas and plays an important role in colorectal cancer progression 42. In the current study, we overexpressed Mist1 and found that E-cadherin was upregulated, while the expression of N-cadherin, Snail and Vimentin were decreased, indicating that Mist1 could inhibit EMT in gastric cancer cells. Next, we investigated the activity of the Wnt/β-catenin pathway and found that the expression levels of β-catenin and several downstream targets, TCF-4, c-Myc, and Cyclin D1, were reduced. Furthermore, TOP/FOPflash luciferase assay revealed that Mist1 overexpression could decrease the transcription activity of β-catenin, suggesting that Mist1 acted as a transcription repressor of β-catenin and overexpression of Mist1 downregulated the Wnt/β-catenin pathway. Notably, when we utilized the Wnt/β-catenin agonist CHIR-99021, which is also known to be a β-catenin activator, changes in the expression of E-cadherin/N-cadherin and β-catenin were reversed, suggesting that Mist1 inhibits EMT in gastric cancer cells via the Wnt/β-catenin pathway.

Valentina *et al*. reported that increased EMT in breast cancer cells was accompanied by a reduction in cell proliferation 43. However, in contrast, in our study, we found that overexpression of Mist1 reversed EMT and inhibited the proliferation of gastric cancer cells, which is consistent with a previous study in the pancreas 28. Furthermore, we found that the Wnt/β-catenin target gene Cyclin D1, an oncogene, was downregulated in Mist1-overexpressing gastric cancer cells. Cyclin D1, a regulatory subunit of the CDK4 or CDK6 complex, is required for the G1/S cell cycle transition, and overexpression of Cyclin D1 may contribute to tumorgenesis 44. Thus, the negative correlation between Mist1 and Cyclin D1 expression might indicate the multiple roles of Mist1 in inhibiting EMT and gastric cancer cell proliferation via Wnt/β-catenin.

However, in this study we could not obtain any gastric cancer cell lines expressing high levels of Mist1 to validate the function of Mist1 by knockdown or knockout cell model, which was a limitation of our study. Additional studies focusing on high Mist1 expressing gastric cells are still needed.

In conclusion, as it plays a critical role in serous secretory cell development and maturation, Mist1 is a marker of gastric chief cells with potential roles in gastric stem cells in the renewal of the gastric mucosa. However, knowledge of Mist1 in gastric carcinogenesis is still limited. In our study, we found that Mist1 was silenced in gastric adenocarcinoma. Furthermore, we identified Mist1 as a tumour suppressor that reduces the proliferation, migration and invasion of gastric cancer cells and inhibits EMT via transcriptional repression of β-Catenin and downregulation of the Wnt/β-Catenin pathway. These findings indicate that Mist1 might be a novel biomarker for early gastric cancer screening and a therapeutic target.

## Supplementary Material

Supplementary figure and table.
