# Supplementary material for: Mist1 Inhibits Epithelial-Mesenchymal Transition in Gastric Adenocarcinoma via Downregulating the Wnt/β-catenin Pathway
Source: J Cancer. 2021 Jun 1;12(15):4574–84. doi: 10.7150/jca.59138 (PMC8210560; doi:10.7150/jca.59138)
Supplement: Supplementary file 1 — Supplementary figure and table. [file jcav12p4574s1.pdf]

Supplementary Figure 1. The morphology changes in Mist1 overexpression gastric cancer cells.

NC

Lv-Mist1

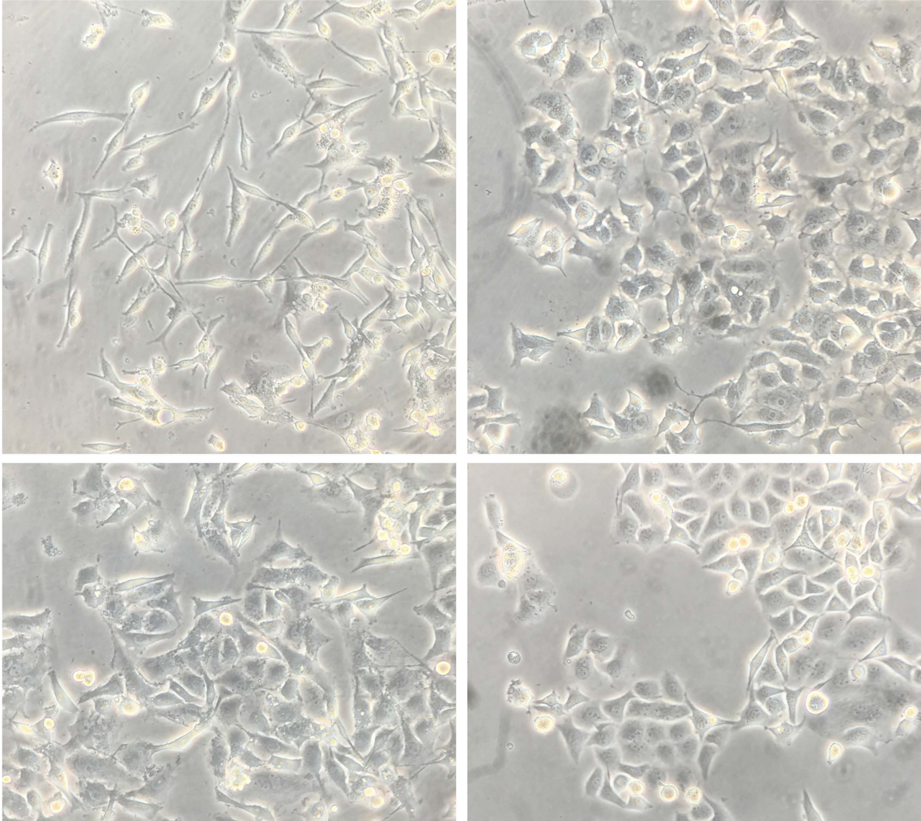

HGC-27 AGS

Supplementary Table 1. The primers used for qPCR amplification

**Primers**	**Sequence**
Mist1	Forward: 5’-CGGATGCACAAGCTAAATAACG-3’
	Reverse: 5’-GCCGTCAGCGATTTGATGTAG-3’
E-cadherin	Forward: 5’-ATTCTGATTCTGCTGCTCTTG-3’
	Reverse: 5’-AGTAGTCATAGTCCTGGTCTT-3’
N-cadherin	Forward: 5’-CGTGAAGGTTTGCCAGTGT-3’
	Reverse: 5’-CAGCACAAGGATAAGCAGGA-3’
β-catenin	Forward: 5’-TGCTGAAGGTGCTATCTGTCTG-3’
	Reverse: 5’-TCCATCCCTTCCTGTTTAGTTG-3’
Snail	Forward: 5’-TTTACCTTCCAGCAGCCCTA-3’
	Reverse: 5’-GACAGAGTCCCAGATGAGCA-3’
Vimentin	Forward: 5’-GGACCAGCTAACCAACGACA-3’
	Reverse: 5’-AAGGTCAAGACGTGCCAGAG-3’
MMP9	Forward: 5’-CGCCAGTCCACCCTTGTG-3’
	Reverse: 5’-TGCCACCCGAGTGTAACCAT-3’
TCF-4	Forward: 5’-TGCAAAGCCGAATTGAAGATCG-3’
	Reverse: 5’- AGAAGGTCCAATGATTCCATGC-3’
c-Myc	Forward: 5’-CCTCCACTCGGAAGGACTATC-3’
	Reverse: 5’-GTGTTCGCCTCTTGACATTCTC-3’
Cyclin D1	Forward: 5’-GCCCTCGGTGTCCTACTTCA-3’
	Reverse: 5’-AAGACCTCCTCCTCGCACTTCT-3’
GAPDH	Forward: 5’-GTCAAGGCTGAGAACGGGAA-3’
	Reverse: 5’-AAATGAGCCCCAGCCTTCTC-3’
